# Cycloaddition reaction of NaN_3_ with nitriles toward the synthesis of tetrazoles catalyzed by a copper complex on boehmite nanoparticles†

**DOI:** 10.1039/d5na00081e

**Published:** 2025-04-16

**Authors:** Arida Jabbari, Bahman Tahmasbi, Elham Mohseni, Mitra Darabi

**Affiliations:** a Department of Chemistry, Qe.C., Islamic Azad University Qeshm Iran aridajabbari@iau.ir; b Department of Chemistry, Faculty of Science, Ilam University P. O. Box 69315516 Ilam Iran b.tahmasbi@ilam.ac.ir

## Abstract

In the present study, the synthesis of boehmite nanoparticles was done using a hydrothermal method using an aluminum source in water solvent. The synthesized boehmite support was modified using (3-iodopropyl)trimethoxysilane (3-IPTMS), and then the modified boehmite was functionalized using a Schiff-base ligand. Finally, copper ions were immobilized on the functionalized boehmite denoted as a boehmite@Schiff-base-Cu nanocatalyst. The synthesized catalyst was identified and confirmed using SEM, FT-IR, TGA, EDXS, WDX, XRD, and BET techniques. The activity of boehmite@Schiff-base-Cu was investigated in preparing 5-substituted tetrazoles using nitrile derivatives and sodium azide, in which short reaction times and high yields were observed in described reactions. Also, the many advantages of the boehmite@Schiff-base-Cu nanocatalyst are ease of operation, compatibility with the environment, its easy separation from the reaction medium, and the ability to reuse it several times without significantly reducing its catalytic activity.

## Abstract 2

A new Schiff-base-Cu complex was immobilized on the modified boehmite nanoparticles and then it was applied as a reusable and selective nanocatalyst in the synthesis of 5-substituted tetrazoles.

## Graphical abstract

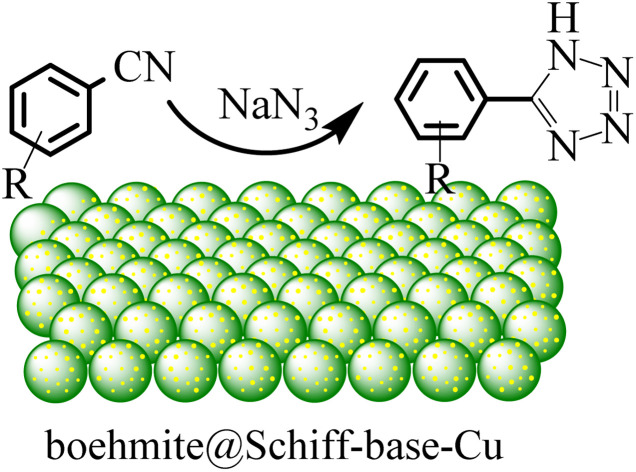

## 1. Introduction

An ideal catalytic system should have good selectivity and activity like homogeneous catalysts. It should also provide ease of recovery and reuse like heterogeneous catalysts. In this regard, in heterogeneous nanocatalysts, as the particle size decreases to the nanoscale, the surface area increases, and a high surface area will be available. Hence, nanocatalysts act as a bridge to the gap between conventional catalysts (homogeneous and heterogeneous). In other words, nanocatalysts provide the advantages of conventional catalysts (homogeneous and heterogeneous) simultaneously.^1,2^ In recent years, the immobilization of homogeneous catalysts on insoluble solid surfaces to recover catalysts from the reaction medium has attracted the attention of researchers in chemistry.^3,4^ The behavior of catalysts immobilized on the support is strongly influenced by the properties of the support. To solve this issue, various nanoheterogeneous supports such as boehmite nanoparticles, mesoporous silica, magnetic nanoparticles, carbon nanotubes, graphene oxide, metal–organic frameworks, zeolites, and ionic liquids have been used to heterogenize homogeneous catalysts.^5–12^ Among the supports mentioned, boehmite nanoparticles have outstanding physical and chemical properties such as high concentrations of surface hydroxyl groups, very high internal surface area, non-toxicity, being inexpensive, and high thermal and chemical stability. For this reason, these nanoparticles are considered one of the most attractive candidates for solid supports.^13–16^ Aluminum oxide has various phases which can be gibbsite [γ-Al(OH)_3_], bayrite [α-Al(OH)_3_], nordstrandite[β-Al(OH)_3_], diaspore[α-AlO(OH)], boehmite[γ-AlOOH] and alumina [Al_2_O_3_]. The most stable of them is α-Al_2_O_3_, and all these phases are intermediate and unstable phases, which after heating finally form an α-Al_2_O_3_ phase.^17–19^ Boehmite is actually one of the phases of aluminum oxide called aluminum oxy-hydroxide AlOOH, which has many applications in the fields of ceramics, petroleum, petrochemicals, and medicine. In addition, boehmite has been used as a catalyst, coating, membrane, optical material, water sweetener, abrasive, absorbent, and vaccine supplement.^20–23^ After alumina phase Al_2_O_3_, boehmite is the most stable aluminum oxide phase. This material starts to convert into an alumina phase in the temperature range of 250–450 °C, during which complete phase change occurs at a temperature of about 450 °C. Therefore, one of the most important applications of boehmite is as a precursor in the preparation of alumina.^24^ Today, there are many methods such as the electrochemical method, hydrothermal method, sol–gel method, and thermal decomposition method to prepare boehmite in nanodimensions. In the meantime, the hydrothermal method is controllable, has a high crystallization ability, and has been used more than other methods.^25–28^ According to the reported studies, boehmite is in the form of cubic structural units (orthorhombic) and the surface of these units contains many hydroxyl groups.^29,30^

Tetrazoles are cyclic materials with a five-membered ring containing 4 nitrogen atoms and 1 carbon atom.^31,32^ Heterocyclic tetrazole derivatives have various applications in the synthesis of other organic compounds and pharmaceutical and biological industries. Because of having low sensitivity to impact and friction, high potential energy of heterocyclic tetrazole derivatives, and high explosion heat, they are good candidates for use in gas and explosives producers. Also, because of having a high percentage of nitrogen, they release a large amount of nitrogen gas after combustion, and for this reason, they have little pollution for the environment and are considered green explosives. In addition, tetrazole compounds play a significant role as ligands in coordination chemistry, so it is important to provide catalytic methods for the synthesis of this group of compounds.^33–35^

We have introduced a new protocol for the catalytic synthesis of tetrazoles using boehmite@Schiff-base@Cu as a heterogeneous nanocatalyst.

## 2. Experimental

### 2.1. Boehmite synthesis

First, 49.6 g of NaOH was dissolved in 50 ml of distilled water and poured into a burette. Then, in a 250 ml beaker, 20 g of Al(NO_3_)_3_·9H_2_O as the aluminum source was dissolved in 30 ml of water and stirred using a stirrer. The sodium hydroxide solution was added drop by drop to the aluminum solution under a mechanical stirrer. Sedimentation was allowed to proceed for twenty minutes with stirring. After sedimentation, the obtained sediment was dispersed using an ultrasonic bath (for 3 h). Then the sediment of the obtained gels was poured into a porcelain crucible and heated for 4 h at 220 °C. The obtained white solid powder is the boehmite crystal. To remove the nitrate impurity, the obtained boehmite was washed with distilled water and dried at 70 °C.^36^

### 2.2. Synthesis of 3-iodopropyltrimethoxysilane

**Scheme 1:**
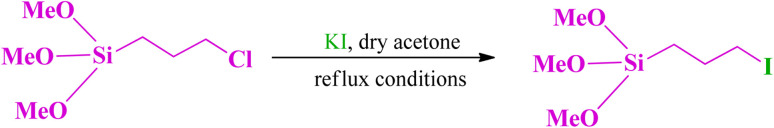
Synthesis of 3-iodopropyltrimethoxysilane (3-IPTMS).

For the synthesis of 3-IPTMS, potassium iodide (0.246 mmol) was first added to dry acetone in a 50 ml flask. Then, the same proportion of 3-chloropropyltrimethoxysilane (0.246 mmol) was refluxed dropwise under a N_2_ atmosphere at 50 °C overnight. After the completion of the reaction, the precipitate of potassium chloride (KI) was filtered, and the 3-iodopropyltrimethoxysilane product, which was in the form of a yellow liquid, was isolated (Scheme 1).^37^

### 2.3. Synthesis of the ligand

**Scheme 2:**
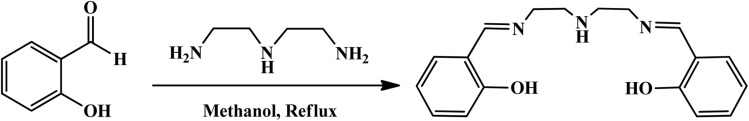
Preparation of a Schiff-base ligand.

For the synthesis of the Schiff-base ligand, salicylaldehyde and di(ethylenetriamine) were used in a ratio of 2 : 1 in methanol solvent. For this purpose, salicylaldehyde (4 mmol) was dissolved in MeOH (methanol) solvent, and then diethylenetriamine (2 mmol) was added dropwise to the mixture. The mixture was stirred under reflux conditions for 4 h. After evaporation of the solvent, the obtained product was dried (Scheme 2).^38^ The final product was purified by recrystallization and characterized using FT-IR spectroscopy.

### 2.4. Synthesis of (PMeOSi)DETA

**Scheme 3:**
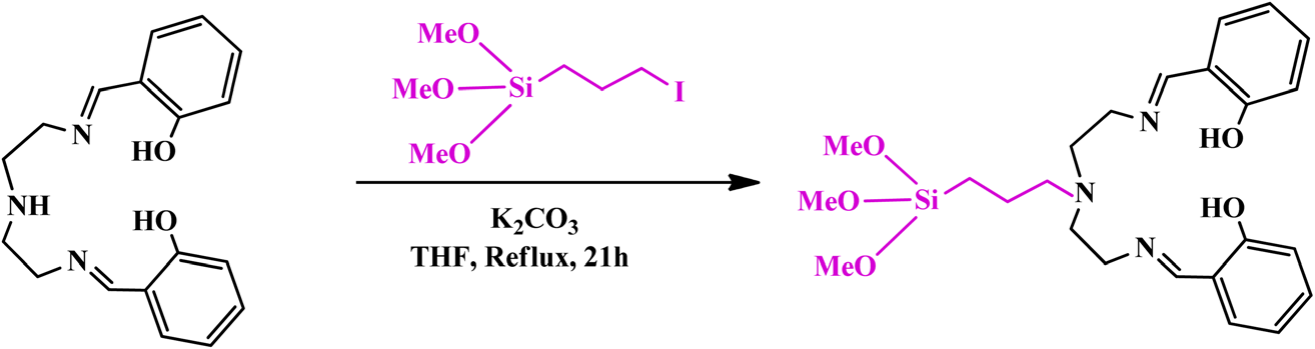
Synthesis of (MeO_3_Si)DETA.

In a 100 ml flask, a mixture of 3-IPTMS (0.310 g) and the Schiff-base ligand (0.330 g) was prepared in THF solvent. Then K_2_CO_3_ (3 mmol) was added to the mixture and refluxed for 21 h at 65 °C. The obtained precipitate was filtered, washed several times with toluene, and dried at 70 °C for 4 h (Scheme 3).^38^

### 2.5. Functionalization of boehmite with (PMeOSi)DETA

**Scheme 4:**
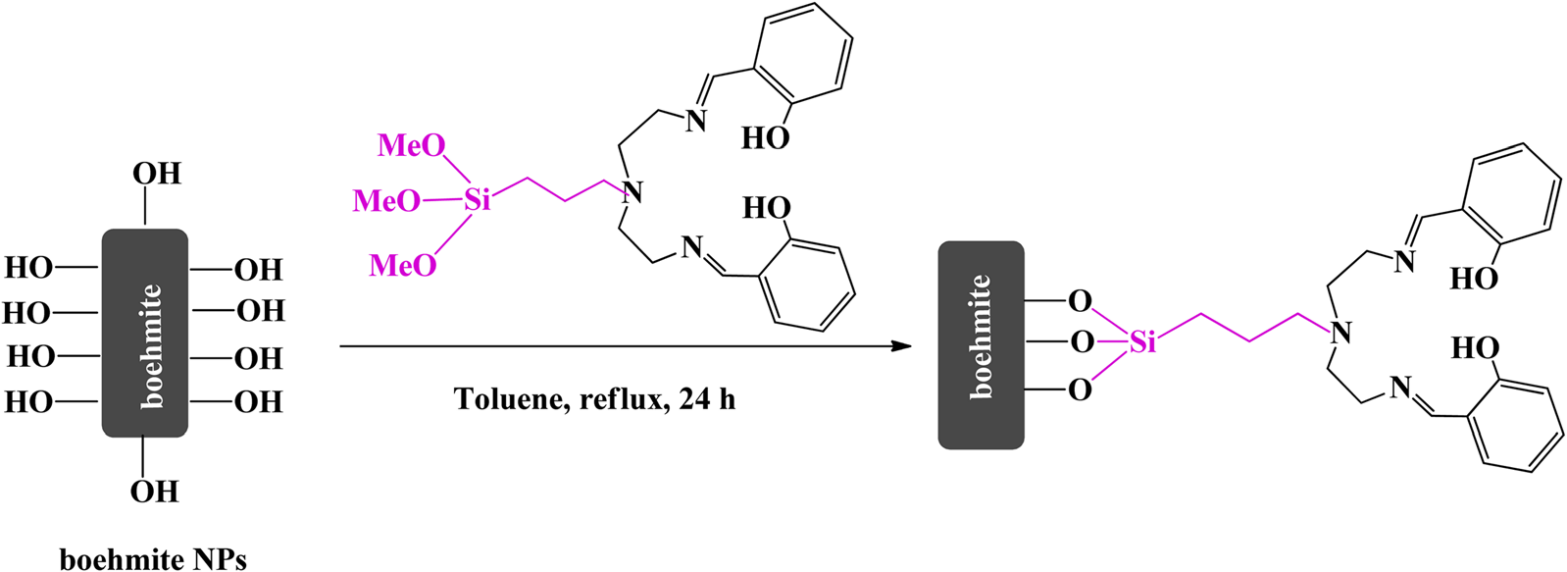
Preparation of boehmite@Schiff-base.

In a 100 ml flask, 1 g of (MeO_3_Si)DETA was dissolved in toluene solvent and then 1.5 g of boehmite support was added to it. The mixture was refluxed for 24 h at 85 °C. After that, the obtained product (boehmite@Schiff-base) was isolated from the mixture using filter paper, washed several times with ethanol, and finally dried at 50 °C (Scheme 4).

### 2.6. Preparation of copper catalysts (boehmite@Schiff-base-Cu)

**Scheme 5:**
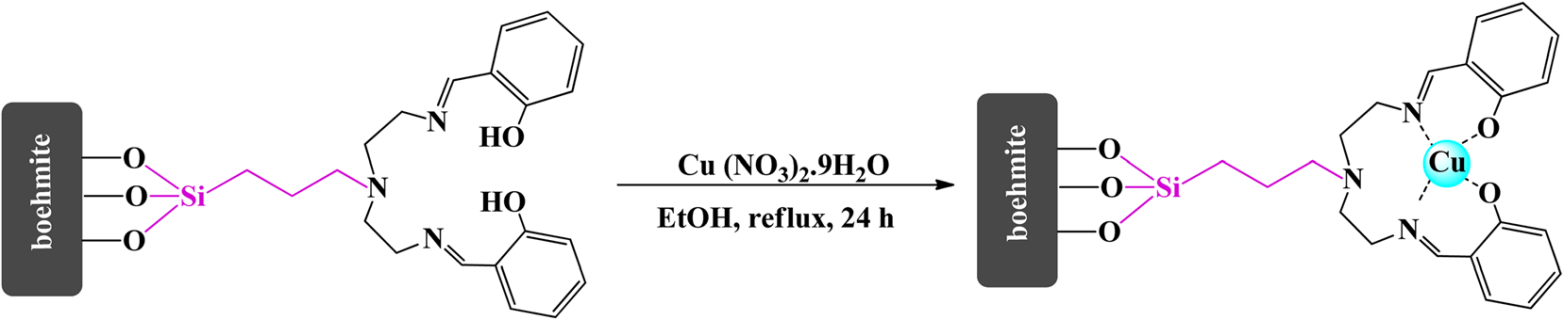
Synthesis of the boehmite@Schiff-base-Cu nanocatalyst.

In the final stage of nanocatalyst synthesis, 1 g of boehmite@Schiff-base and 3 mmol of Cu(NO_3_)_2_·9H_2_O were dissolved in ethanol solvent. The reaction mixture was refluxed for 24 h. After the reaction finished, the synthesized nanocatalyst (boehmite@Schiff-base-Cu) was filtered and washed with distilled water and ethanol. Finally, it was dried at 60 °C (Scheme 5).

### 2.7. Synthesis of tetrazoles

**Scheme 6:**
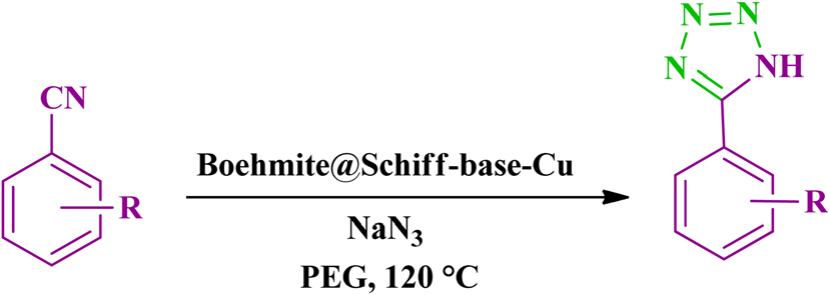
Synthesis of tetrazoles catalyzed by the boehmite@Schiff-base-Cu nanocatalyst.

In a 25 ml round bottom flask, a suspension was formed with a mixture of 35 mg of boehmite@Schiff-base-Cu nanocatalyst, 1 mmol of nitrile, 1.2 mmol of NaN_3_ (sodium azide), and a sufficient amount of PEG (2 ml) at a temperature of 120 °C under a magnetic stirrer. The progress of the reaction was checked by TLC in a mixture solvent of *n*-hexane and acetone at a ratio of (4 : 1). After the end of the reaction, the nanocatalyst was separated using filter paper. Then, 10 ml of 4N HCl and 7 ml of ethyl acetate were added to the mixture, and the organic phase extracted using a decanter was dried at ambient temperature (Scheme 6).

### 2.8. Spectral data

#### 2.8.1. 4-(1*H*-tetrazol-5-yl)benzonitrile

^1^H NMR (250 MHz, DMSO-d6): *δ*_H_ = 8.21–8.18 (d, *J* = 7.5 Hz, 2H), 8.07–8.04 (d, *J* = 7.5 Hz, 2H) ppm.

#### 2.8.2. 2-(1*H*-tetrazol-5-yl)phenol

^1^H NMR (250 MHz, DMSO): *δ*_H_ = 13.07 (br, 1H), 8.05–7.89 (m, 1H), 7.42–7.30 (m, 1H), 7.13–6.91 (m, 2H) ppm.

## 3. Results and discussion

As indicated in Scheme 1, we synthesized the boehmite@Schiff-base-Cu nanocatalyst for the first time and we investigated its catalytic performance in the synthesis of tetrazoles from various nitrile derivatives. The prepared nanocatalyst was identified by some techniques such as BET (Brunauer–Emmett–Teller), scanning electron microscopy (SEM), energy-dispersive X-ray spectroscopy (EDXS), thermogravimetric analysis (TGA), wavelength-dispersive X-ray spectroscopy (WDX), X-ray diffraction (XRD) and Fourier transform-infrared spectroscopy (FT-IR).

### 3.1. N_2_ adsorption–desorption isotherm studies

**Fig. 1:**
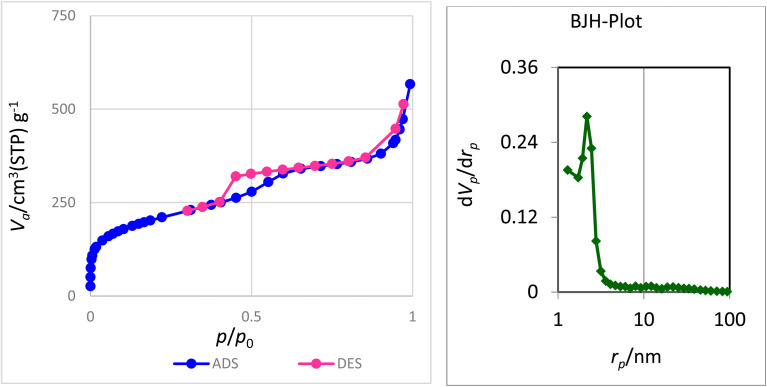
N_2_ adsorption–desorption isotherms and BJH-plot of the boehmite@Schiff-base-Cu nanocatalyst.

The nitrogen adsorption–desorption technique was used to determine the structural characteristics and examine the surface of nanocatalyst boehmite@Schiff-base-Cu. Fig. 1 shows the nitrogen adsorption–desorption analysis at 120 °C and the pore size distribution plot corresponding to the N_2_ adsorption–desorption for boehmite@Schiff-base-Cu. As it is clear in this figure, this diagram is a type IV isotherm (definition by IUPAC) in the region of relative pressure (*P*/*P*_0_) between 0.4 and 0.8, which is characteristic of mesoporous compounds.^39,40^ The structural parameters of nanocatalyst boehmite@Schiff-base-Cu, such as the average pore diameter, surface area, and total pore volume, are listed in Table 1. The average pore diameter, total pore volume, and specific surface area of the boehmite@Schiff-base-Cu nanocatalyst are 4.6495 nm, 0.8601 cm^3^, and 739.96 m^2^ g^−1^, respectively. Also, there is only one sharp peak at 2.3 nm as observed from the pore size distribution plot derived from this isotherm.

**Table 1:** Structural and textural parameters of boehmite@Schiff-base-Cu samples

Sample	*S*_BET_ (m^2^ g^−1^)	Pore diameter (nm)	Proe volume (cm^3^)
boehmite@Schiff-base-Cu	739.96	4.6495	0.8601

### 3.2. Thermogravimetric analysis studies

**Fig. 2:**
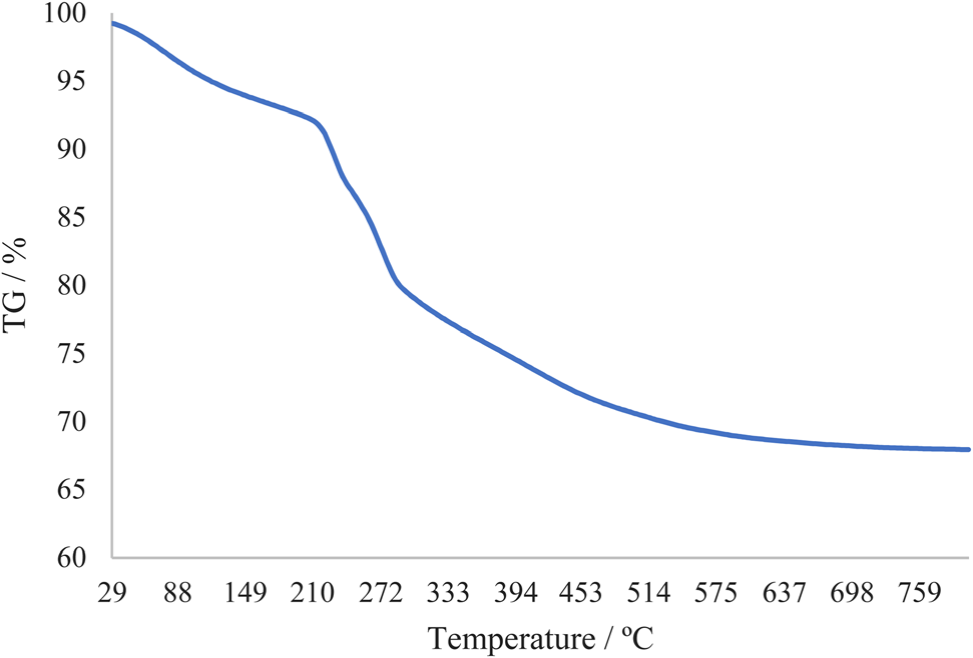
TGA curves of the boehmite@Schiff-base-Cu nanocatalyst.

The thermal stability of catalyst boehmite@Schiff-base-Cu was investigated through thermogravimetric analysis. This TGA analysis was done in the heat range of 29 to 800 °C. The TGA diagram of boehmite@Schiff-base-Cu is shown in Fig. 2. The first weight loss (about 8%) at low temperatures is related to the evaporation of solvents. As indicated, except for the evaporation of the solvent, no weight loss occurred up to 210 °C, meaning that the boehmite@Schiff-base-Cu catalyst is stable up to 210 °C. The curve shows a weight loss of about 22% from the temperature range of 210–450 °C, which indicates the well stabilization of the copper complex on the boehmite nanoparticles.

### 3.3. SEM photographs

**Fig. 3:**
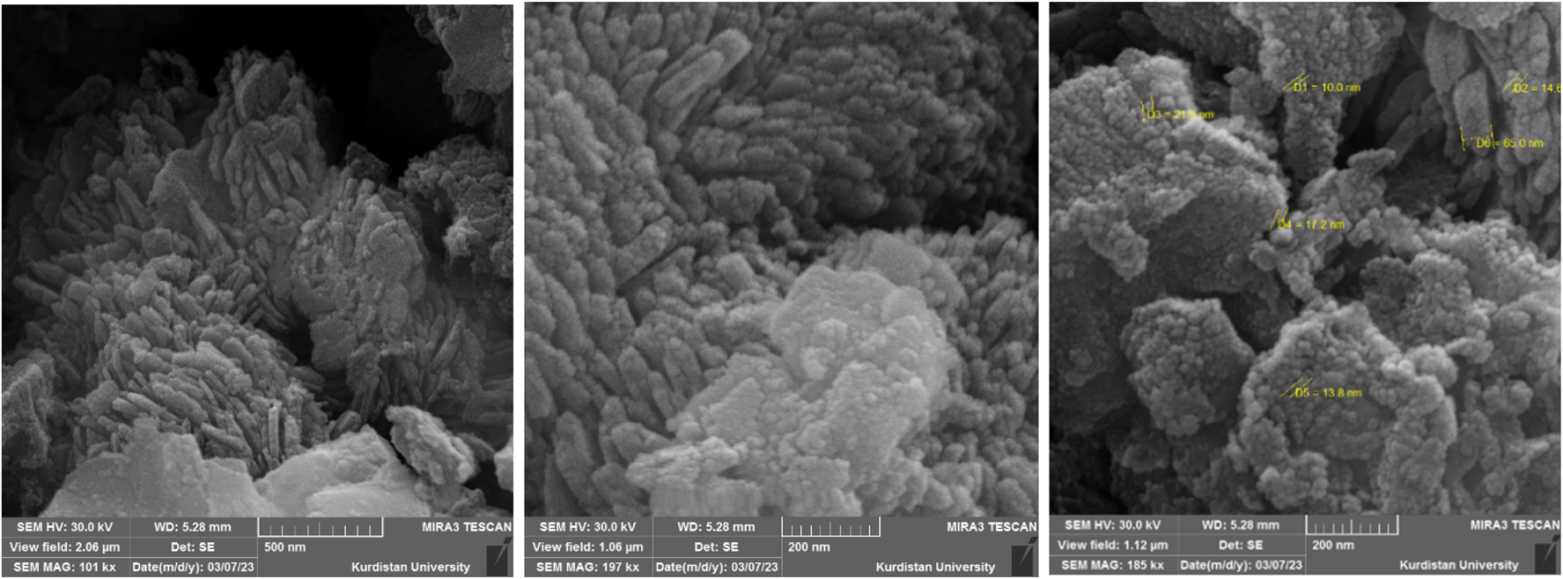
SEM images of the boehmite@Schiff-base-Cu nanocatalyst.

The particle size and morphology of the boehmite@Schiff-base-Cu nanocatalyst were investigated by SEM. Fig. 3 shows the SEM images of the nanocatalyst. As shown in the images, this catalyst has been synthesized as cubic structural units (orthorhombic) with a uniform size between 10 and 25 nm.

### 3.4. Energy dispersive X-ray analysis and elemental mapping

**Fig. 4:**
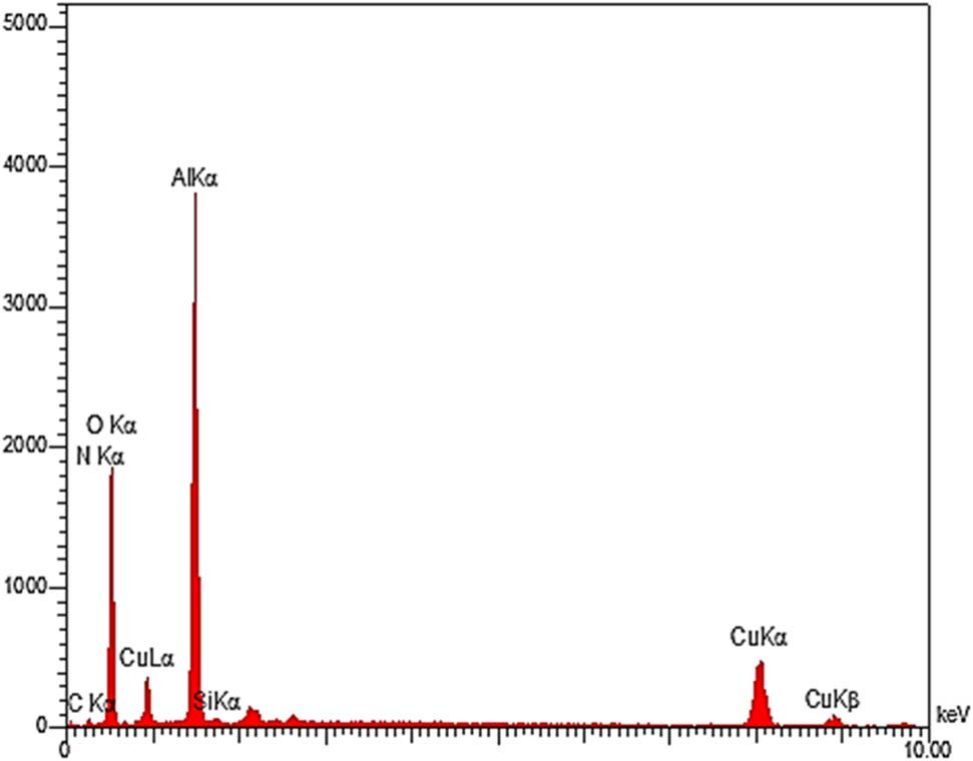
The EDS spectra of the boehmite@Schiff-base-Cu nanocatalyst.

**Fig. 5:**
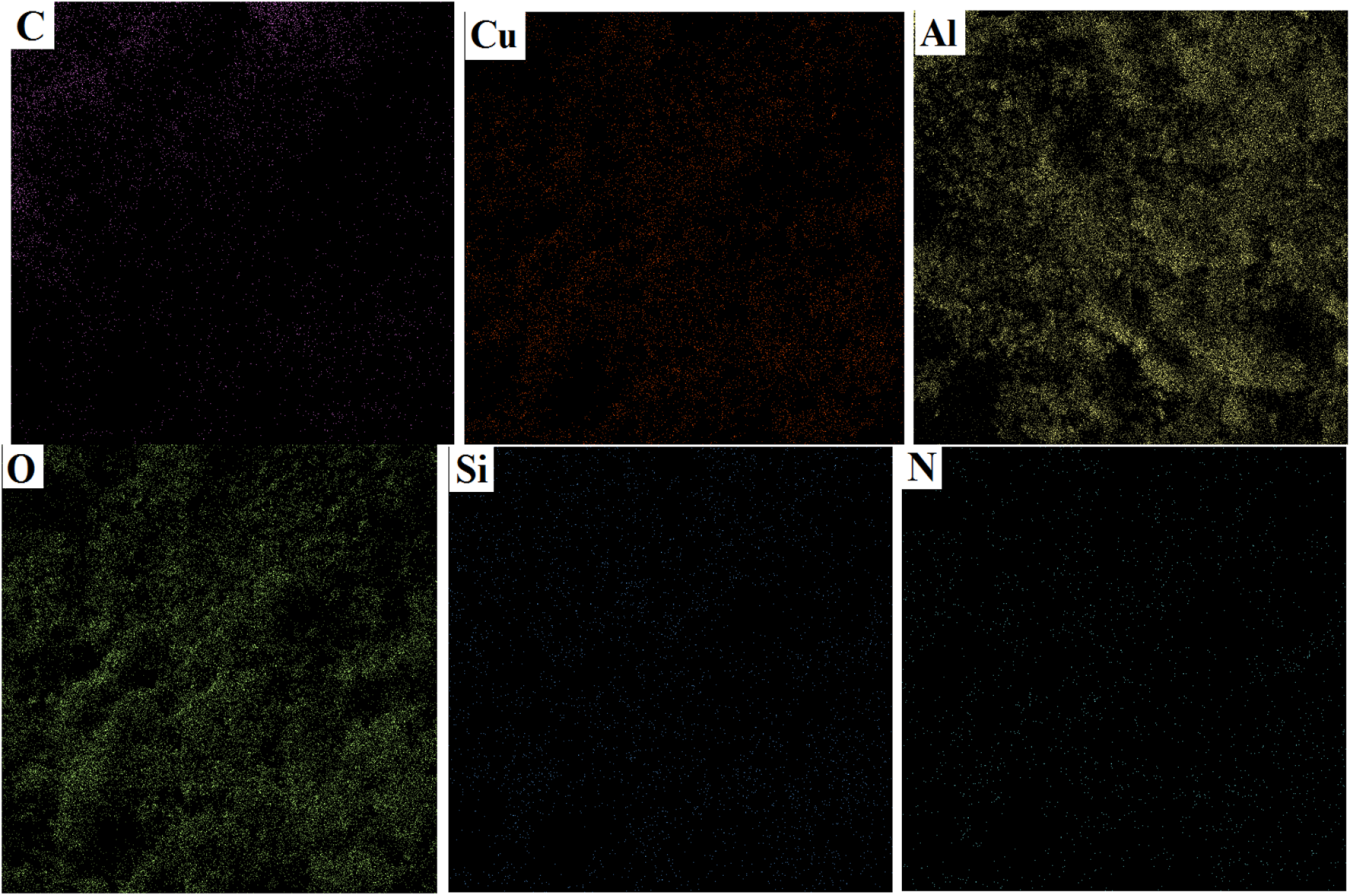
Elemental mapping images of the boehmite@Schiff-base-Cu nanocatalyst.

The EDS analysis is a method to determine the elemental composition of a sample. The EDS technique was used to determine the quality of the elements in the structure of boehmite@Schiff-base-Cu. The diagram obtained from this analysis is shown in Fig. 4. The obtained results showed that O, Al, C, Si, N, and Cu elements are present in the prepared nanocatalyst structure. This evidence confirms the successful synthesis of this nanocatalyst. Fig. 5 shows the distribution of the elements in boehmite@Schiff-base-Cu. In these images, the presence of aluminum, silicon, oxygen, carbon, nitrogen, and copper elements is visible. Also, the distribution of copper on the support surface was confirmed by this elemental analysis.

### 3.5. FT-IR spectra

**Fig. 6:**
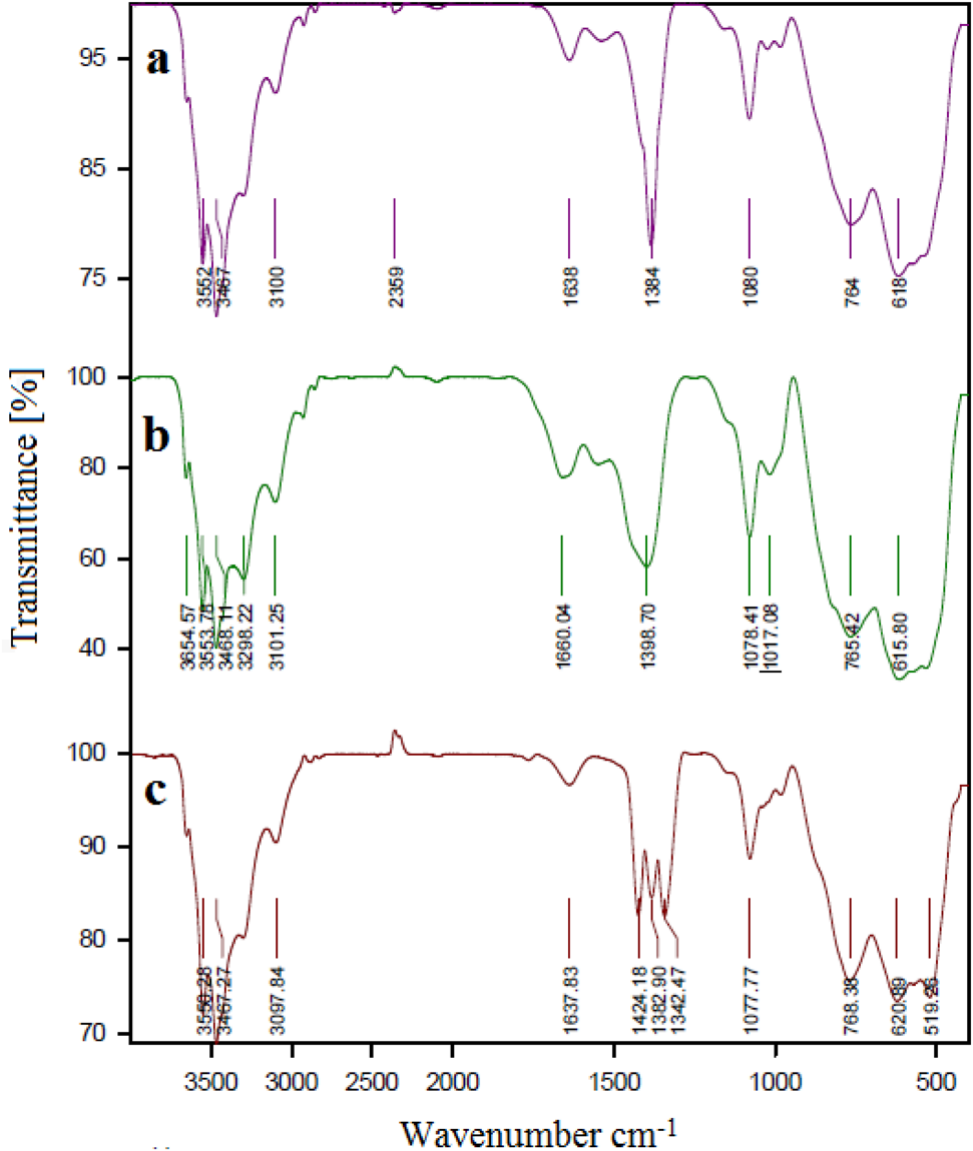
FT-IR spectra of (a) boehmite, (b) boehmite@Schiff-base and (c) boehmite@Schiff-base-Cu nanocatalyst.

FTIR spectroscopy makes it possible to verify the functional group in the structure of the synthesized catalyst. Fig. 6 shows the FT-IR spectra of (a) boehmite support, (b) boehmite@Schiff-base, and (c) boehmite@Schiff-base-Cu. In spectrum a, the peaks at 3467 cm^−1^ (symmetric) and 3552 cm^−1^ (asymmetric) displayed in the spectrum of boehmite nanoparticles are related to the vibrations of surface O–H bonds that are attached to the surface of boehmite nanoparticles.^41^ The peaks appearing in all IR-FT spectra in the regions of 618 cm^−1^ and 764 cm^−1^ are attributed to the Al–O bond vibrations in the boehmite core.^42^ Also, the peak shown at 1638 cm^−1^ is related to the bending vibration of hydrogen bonds of surface hydroxyl groups.^43^ In the IR spectra of boehmite with a Schiff-base functionalized ligand (Fig. 6b), stretching vibrational bands C=C, C=N, and O–H were observed at 1660, 1394, and >3000 cm^−1^ respectively and the peak shown at 1080 cm^−1^ is attributed to the vibration of Si–O. The FTIR spectrum of the catalyst (Fig. 6c) shows the C=N stretching vibrational band at a lower wave number (1637 cm^−1^). Based on these data, copper metal ions of C=N groups were coordinated.^38^

### 3.6. X-ray diffraction

**Fig. 7:**
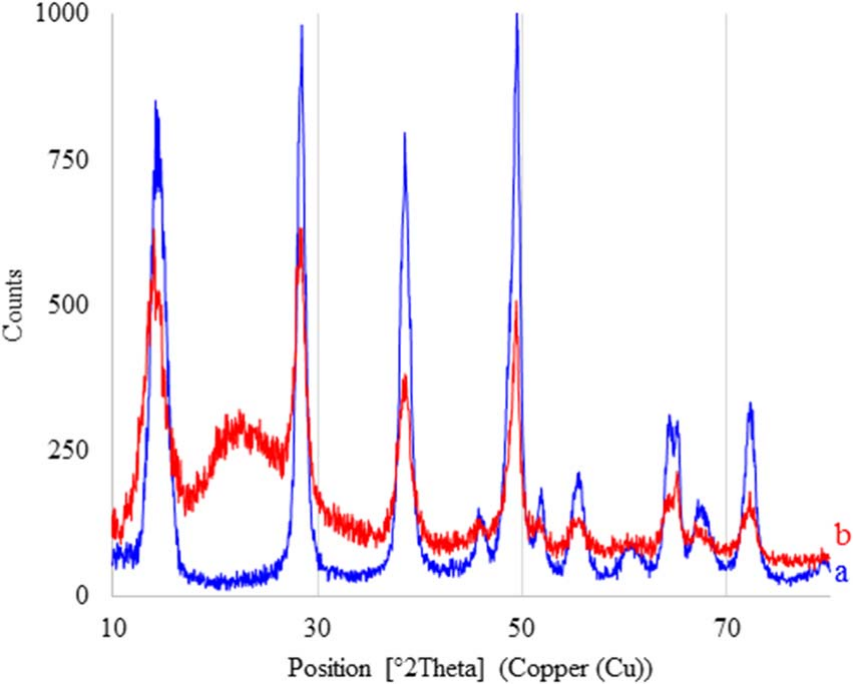
XRD patterns of (a) boehmite and (b) boehmite@Schiff-base-Cu nanocatalyst.

The normal XRD patterns of boehmite and the boehmite@Schiff-base-Cu nanocatalyst are shown in Fig. 7. The XRD diffraction of boehmite (Fig. 7a) exhibits a series of peaks at different 2*θ* positions at 14.32° (0 2 0), 28.47° (1 2 0), 38.52° (0 3 1), 45.72° (1 3 1), 49.47° (0 5 1), 51.87° (2 0 0), 55.72° (1 5 1), 60.70° (0 8 0), 64.42° (2 3 1), 65.27° (0 0 2), 67.97° (1 7 1), and 72.52° (2 5 1), which are related to the standard pattern of boehmite nanoparticles in the orthorhombic unit cell (JCPDS-no. 00-049-0133 and JCPDS-no. 01-074-1895).^44–49^ All these peaks are also clearly observed in the XRD patterns of the boehmite@Schiff-base-Cu nanocatalyst, indicating that the crystal structure of boehmite nanoparticles remained stable after functionalization and stabilization of the copper complex in orthorhombic cells (Fig. 7b).

Moreover, a broad peak of the 2*θ* value at the 18–26° position is related to the coated silica on boehmite nanoparticles,^3,50^ which is not observed in the XRD pattern of unmodified boehmite nanoparticles. This peak indicates that boehmite nanoparticles were successfully modified with (3-iodopropyl)trimethoxysilane.

### 3.7. Catalytic studies

Various experiments were conducted to investigate the boehmite@Schiff-base-Cu nanocatalyst and to optimize the conditions in the reaction related to 4-nitrobenzonitrile, including the type of solvent, the amount of the catalyst, and the temperature. To obtain optimal conditions for the synthesis of tetrazoles, the reaction of 4-nitrobenzonitrile (1 mmol) with sodium azide (1.2 mmol) in the vicinity of the boehmite@Schiff-base-Cu catalyst was selected as a sample reaction. The results related to the effect of different parameters on this reaction were investigated, which are summarized in Table 2. At first, the reaction was investigated in the presence of different amounts of the boehmite@Schiff-base-Cu catalyst; according to the results in the table, the shortest time and the highest yield were obtained in the presence of 35 mg of boehmite@Schiff-base-Cu nanocatalyst. Then the effects of different solvents such as DMSO, PEG-400, 1,4-dioxane, toluene, ethanol, and H_2_O were compared, and it was found that PEG-400 solvent provides the best results. Finally, the effect of temperature on the reaction of the model was investigated. The best results with excellent yield and low reaction time were obtained in PEG-400 solvent (as green solvent), for an amount of 35 mg of boehmite@Schiff-base-Cu nanocatalyst, and at a temperature of 120 °C (Table 2).

**Table 2:** Effect of various parameters on the formation of tetrazoles in the existence of the boehmite@Schiff-base-Cu catalyst

Entry	Solvent	Temp. (°C)	Catalyst (mg)	Time (min)	Yield (%)
1	PEG	120	40	60	95
2	PEG	120	35	85	95
3	PEG	120	30	90	70
4	PEG	100	35	170	Trace
5	PEG	120	—	120	N.R
6	H_2_O	100	35	120	Trace
7	EtOH	77	35	120	Trace

After obtaining the conditions, the [3 + 2] cycloaddition reaction of nitrile derivatives and NaN_3_ was examined for the preparation of several types of tetrazoles (Table 3). All nitriles, including electron- (donating or accepting) functional groups, became related tetrazoles in the presence of boehmite@Schiff-base-Cu. Significantly, boehmite@Schiff-base-Cu exhibits a good homoselectivity in the tetrazole synthesis, when two similar types of cyano-substituted groups are present in two quite same positions of the benzonitrile ring, *e.g.* phthalonitrile and terephthalonitrile, that only mono-cycloaddition was observed (Table 3, entries 2 and 4). The homoselectivity of the boehmite@Schiff-base-Cu catalyst in the synthesis of tetrazoles was confirmed with ^1^H NMR spectroscopy, as ^1^H NMR (250 MHz, DMSO-d6): *δ*_H_ = 8.21–8.18 (d, *J* = 7.5 Hz, 2H), 8.07–8.04 (d, *J* = 7.5 Hz, 2H) ppm.

**Table 3:** Synthesis of some tetrazoles catalyzed by boehmite@Schiff-base-Cu

Entry	Substrate	Product	Time (min)	Yield (%)
1	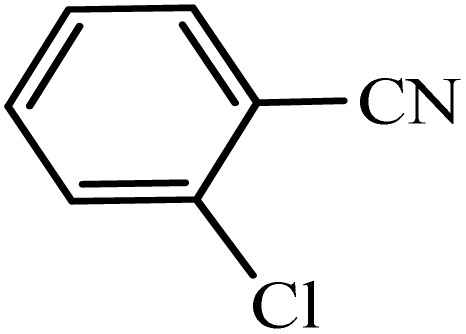	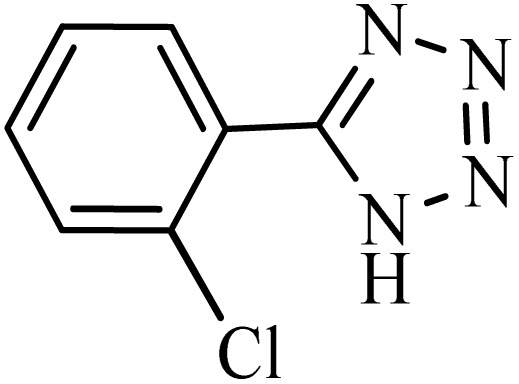	100	90
2	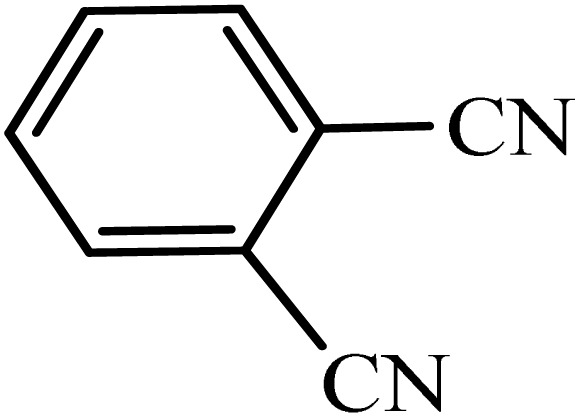	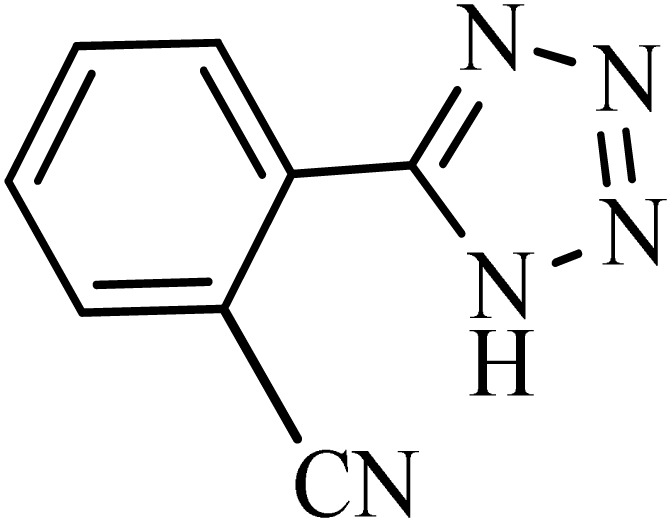	120	95
3	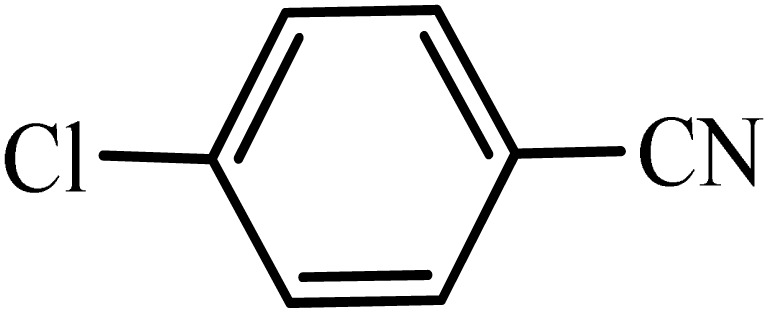	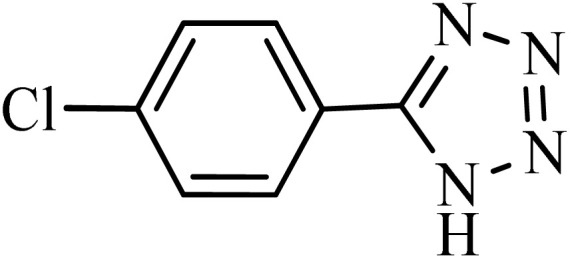	200	70
4	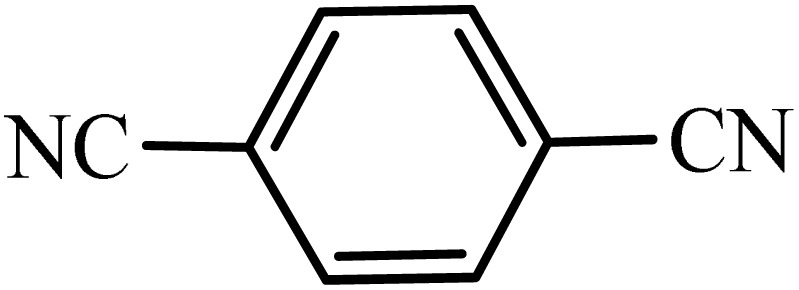	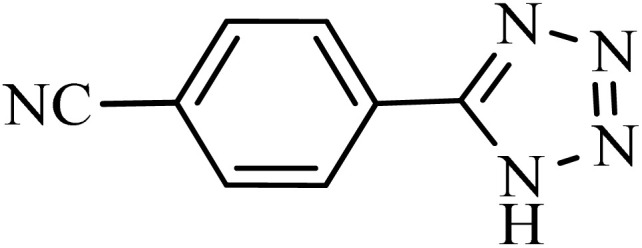	70	95
5	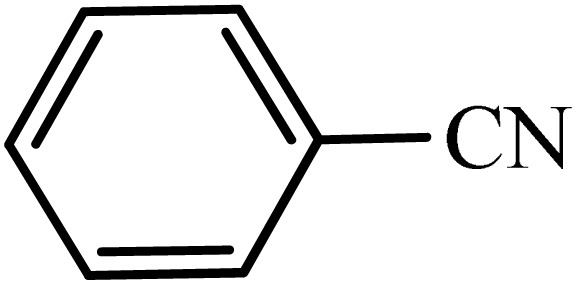	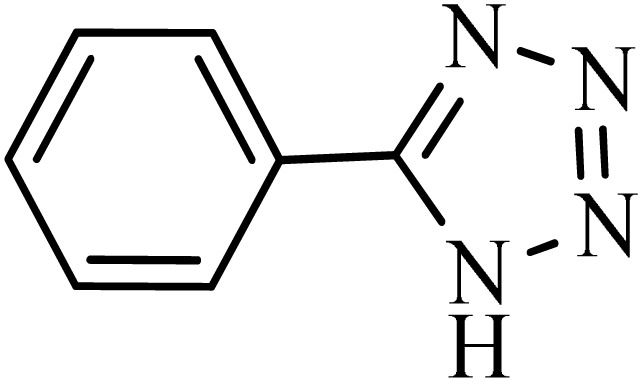	135	85
6	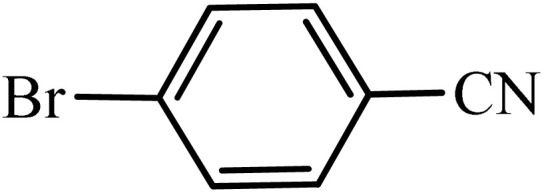	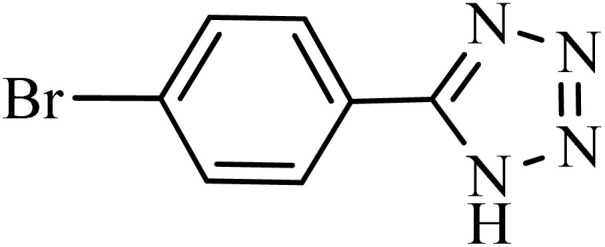	180	78
7	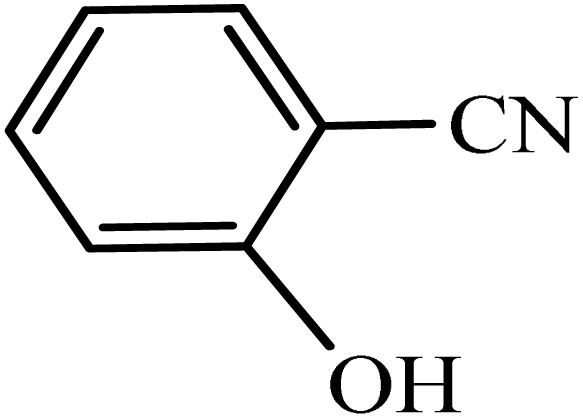	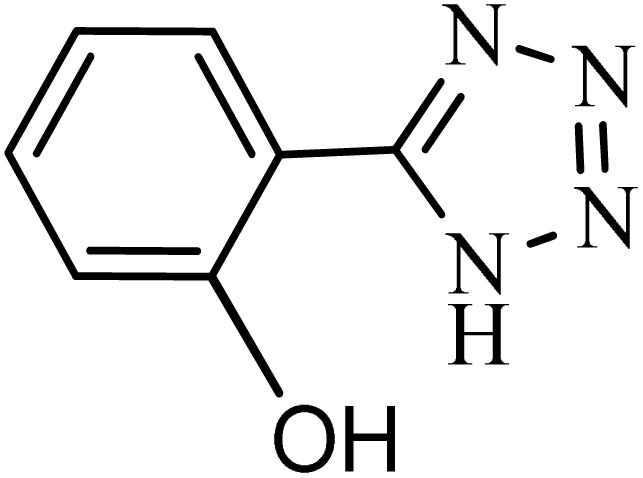	100	98
8	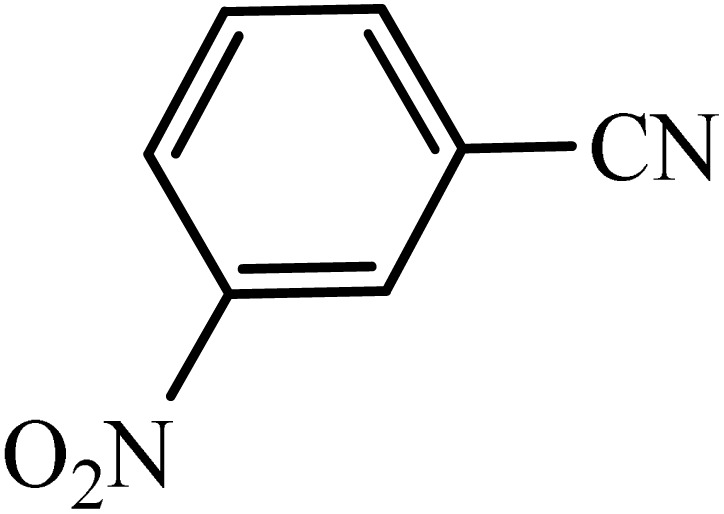	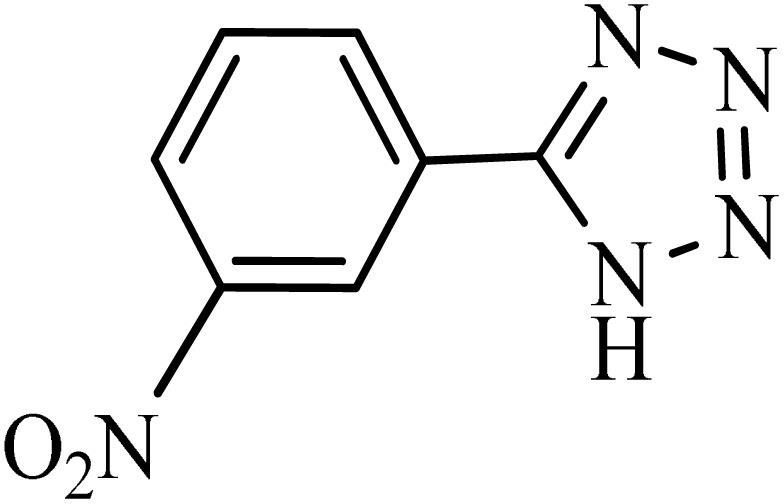	100	85
9	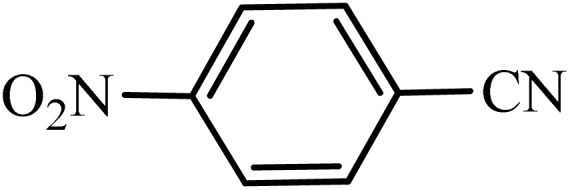	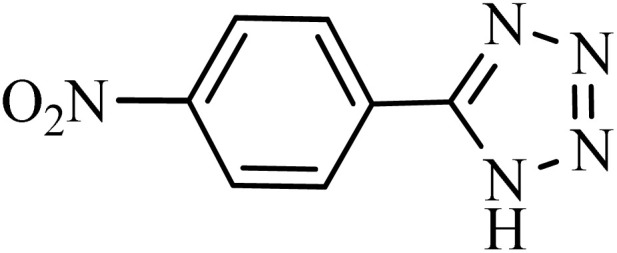	85	95

**Scheme 7:**
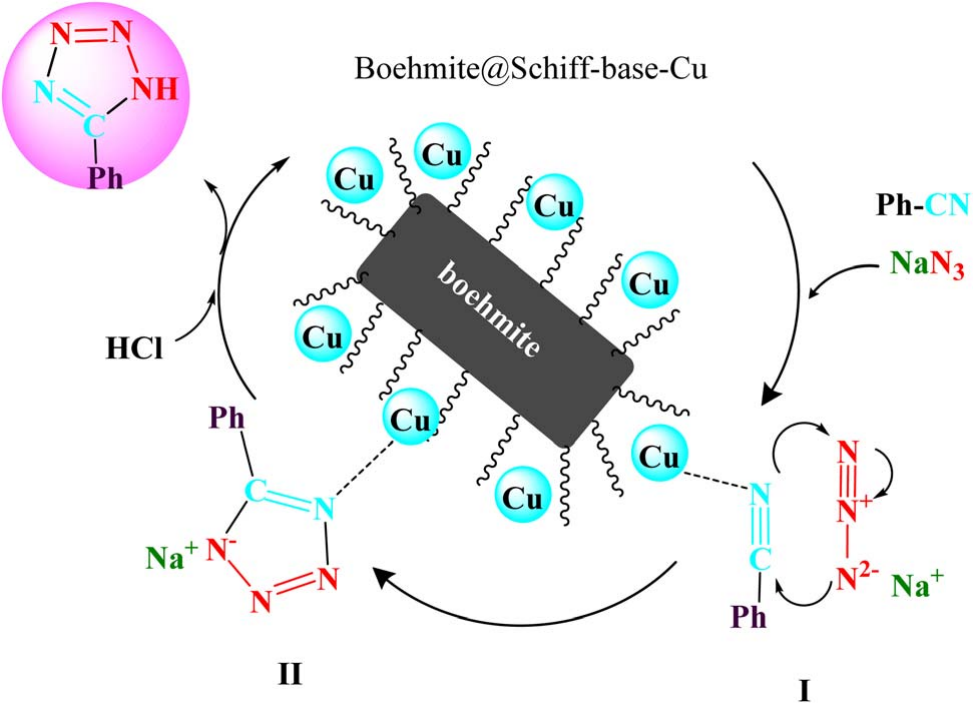
A cyclic mechanism for the formation of tetrazoles catalyzed by boehmite@Schiff-base-Cu.

A reaction mechanism for the formation of tetrazoles catalyzed by boehmite@Schiff-base-Cu is shown in Scheme 7.^51–53^ In this suggested mechanism, at first, the nitrile group becomes susceptible to nucleophilic attack with the interaction of the cyano-functional group with the boehmite@Schiff-base-Cu catalyst. At this stage, intermediate I is formed. Then, intermediate II is formed through the [3 + 2] cycloaddition reaction with NaN_3_ and intermediate I as a sodium salt form. In the final stage, the salt form of the intermediate II is converted to a target molecule tetrazole compound through HCl addition in the work-up step.

### 3.8. Reusability of the catalyst

**Fig. 8:**
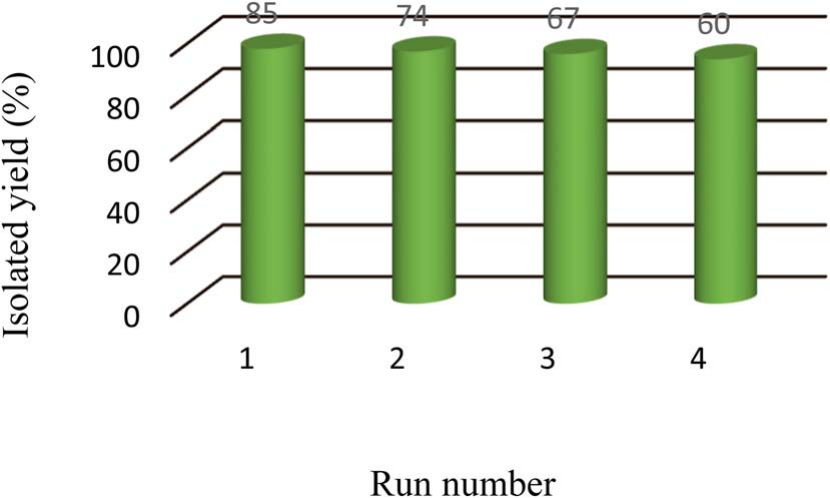
Recyclability study of the boehmite@Schiff-base-Cu nanocatalyst.

To investigate the recovery and reusability of the boehmite@Schiff-base-Cu nanocatalyst in the synthesis of tetrazoles under optimal conditions, the synthesis of 5-phenyl-1*H*-tetrazole was selected as a model reaction. After the end of the reaction in each cycle, the catalyst was separated using centrifugation and washed several times with HCl 4N and hot ethyl acetate and then it was reused in the next cycle after drying. The catalyst was recycled in 4 periods without a significant decrease in its activity. In Fig. 8, the activity results of the recycled boehmite@Schiff-base-Cu nanocatalyst are shown in the form of a diagram.

To investigate the heterogeneous nature of the boehmite@Schiff-base-Cu nanocatalyst, a hot filtration test was carried out based on a published article.^28^ In this regard, in the synthesis of 5-phenyl-1*H*-tetrazole, the catalyst was removed after finishing the reaction and then the exact amount of probabilistic leached copper in the filtered reaction media was calculated by AAS analysis. In this analysis, a notable amount of leached copper was not detected. These results indicate that copper is not leached from the boehmite@Schiff-base-Cu catalyst and that this catalyst has a heterogeneous nature.

**Fig. 9:**
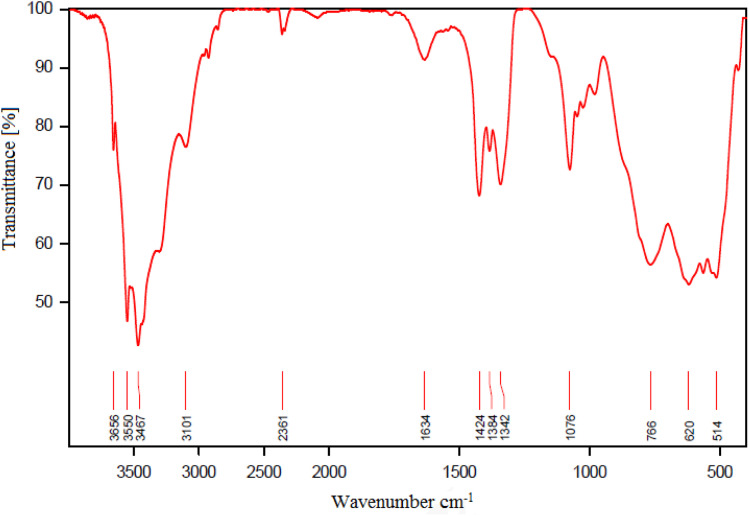
FT-IR spectra of recovered boehmite@Schiff-base-Cu.

The structure of the recovered boehmite@Schiff-base-Cu nanocatalyst was identified using FT-IR analysis (Fig. 9). As it is clear from the FT-IR spectrum, there is no significant change in the recycled nanocatalyst compared to the original catalyst. These results indicate that the boehmite@Schiff-base-Cu nanocatalyst is stable under the reaction conditions for the formation of tetrazoles.

### 3.9. Comparison of the catalyst

The activity of the boehmite@Schiff-base-Cu catalyst was evaluated in comparison with other previously reported catalysts in the literature (Table 4). According to the results given in Table 4, the synthesis reaction of 4-(1*H*-tetrazol-5-yl)benzonitrile has been carried out in PEG-400 as a green solvent with low reaction time and high yield of the product. The results in Table 4 prove the superiority of the boehmite@Schiff-base-Cu catalyst in terms of efficiency or reaction time compared to other catalysts in the literature.

**Table 4:** Comparison of the boehmite@Schiff-base-Cu catalyst for the synthesis of 4-(1*H*-tetrazol-5-yl)benzonitrile with previous catalysts

Entry	Catalyst	Solvent	Time (min)	Yield (%)
1	Boehmite@Schiff-base-Cu	PEG	70	95 [This work]
2	Fe_3_O_4_@SBTU@Ni(ii)	PEG	7 h	94 ref. (54)
3	Cu(ii) immobilized on Fe_3_O_4_@SiO_2_@l-histidine	PEG	90	95 ref. (55)
4	FeCl_3_–SiO_2_	PEG	20 h	80 ref. (56)
5	Fe_3_O_4_-adenine-Zn	PEG	140	91 ref. (57)
6	BNPs@Cur-Ni	PEG	120	88 ref. (58)
7	Boehmite@SiO_2_@Tris-Cu(i) NPs	PEG	110	89 ref. (59)
8	CdCl_2_	PEG	9 h	79 ref. (60)
9	Fe_3_O_4_@boehmite	PEG	24 h	92 ref. (61)

## 4. Conclusion

In this research, a new and green nanocatalyst (boehmite@Schiff-base-Cu) was prepared, whose catalytic activity was investigated in the important synthesis of tetrazoles using nitrile derivatives and NaN_3_, in PEG-400 solvent at 120 °C. This nanocatalyst was identified by SEM, FT-IR, TGA, EDXS, WDX, XRD, and BET. The advantages of tetrazole synthesis in the presence of boehmite@Schiff-base-Cu include short reaction time, high yield of products, stability of the catalyst, and inexpensive and availability of reagents. Also, the use of this nanocatalyst in this work compared to other catalysts has advantages such as low toxicity, transportation, easy storage, weighing and utilization, simple preparation of the catalyst from cheap and available raw materials, high catalytic activity due to the increased surface area, stability, and recyclability and reusability of the catalyst.

## Conflicts of interest

There are no conflicts to declare.

## Supplementary Material

NA-007-D5NA00081E-s001

## Data Availability

The data that support the findings of this study are available in the article file and ESI.†
