# Supplementary material for: Cycloaddition reaction of NaN3 with nitriles toward the synthesis of tetrazoles catalyzed by a copper complex on boehmite nanoparticles
Source: Nanoscale Adv. 2025 Apr 16;7(12):3664–75. doi: 10.1039/d5na00081e (PMC12057638; doi:10.1039/d5na00081e)

Supplementary Information (SI) for Nanoscale Advances.
This journal is © The Royal Society of Chemistry 2025

Cycloaddition reaction of NaN_3 with nitriles toward the synthesis of tetrazoles catalyzed by copper complex on boehmite nanoparticles

Arida Jabbari*[,1], Bahman Tahmasbi[2], Elham Mohseni[2], Mitra Darabi[2]

Abstract

In the present study, the synthesis of boehmite nanoparticles was done using a hydrothermal method using an aluminum source in water solvent. The synthesized boehmite support was modified using 3-IPTMS organosilane ((3-Iodopropyl)trimethoxysilane), and then the modified boehmite was functionalized by a Schiff-base ligand. Finally, the copper ions were immobilized on the functionalized boehmite denoted as boehmite@Schiff-base-Cu nanocatalyst. The synthesized catalyst was identified and confirmed using SEM, FT-IR, TGA, EDXS, WDX, XRD, and BET techniques. The activity of boehmite@Schiff-base-Cu was investigated in preparing 5-substituted tetrazoles using nitrile derivatives and sodium azide, in which short reaction times and high yields were observed in described reactions. Also, the many advantages of boehmite@Schiff-base-Cu nanocatalyst are ease of operation, compatibility with the environment, easy separation of the catalyst from the reaction medium, and the ability to reuse it several times without significantly reducing its catalytic activity.

Keywords: boehmite, nanocatalyst, copper complex, 5-substituted tetrazoles, Schiff-base.

¹H NMR spectral data

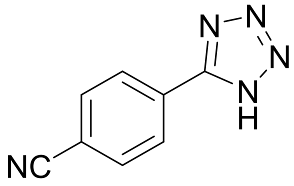

4-(1H-tetrazol-5-yl)benzonitrile

^{1}H NMR (250 MHz, DMSO-d6): δ_H= 8.21-8.18 (d, *J*= 7.5 Hz, 2H), 8.07-8.04 (d, *J*= 7.5 Hz, 2H) ppm.

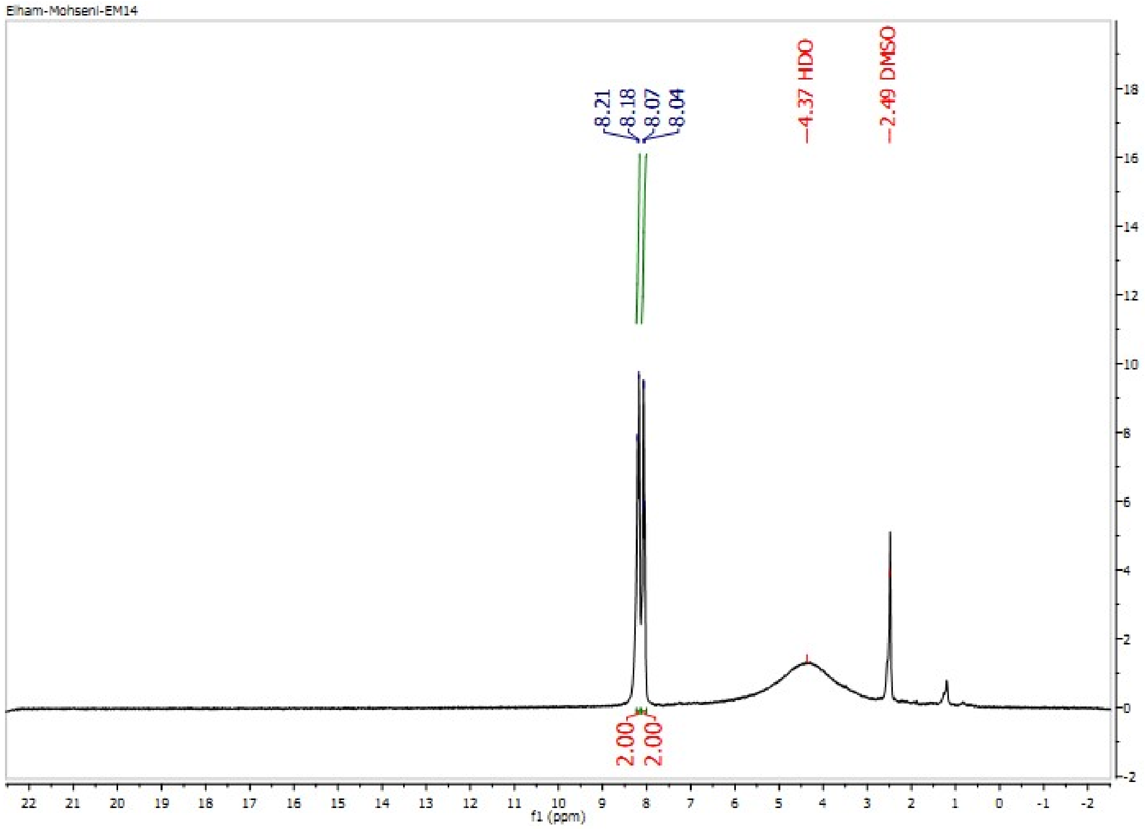

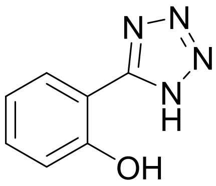

2-(1H-tetrazol-5-yl)phenol

^{1}H NMR (250 MHz, DMSO): δ_H= 13.07 (br, 1H), 8.05-7.89 (m, 1H), 7.42-7.30 (m, 1H), 7.13-6.91 (m, 2H) ppm.

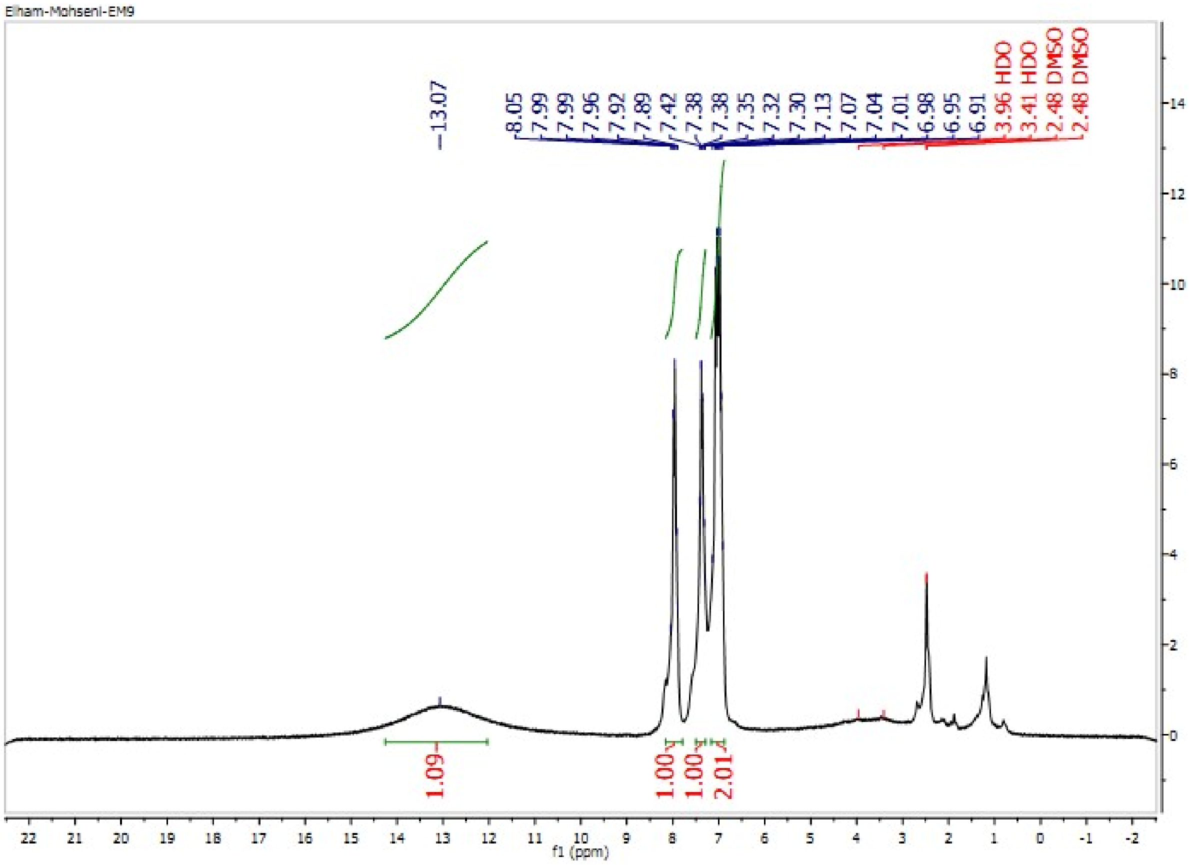

Supplement: NA-007-D5NA00081E-s001 [file NA-007-D5NA00081E-s001.pdf]
